# A single-center pilot randomized controlled trial of atorvastatin loading for preventing ischemic brain damage after carotid artery stenting

**DOI:** 10.3389/fnagi.2022.1066316

**Published:** 2022-12-23

**Authors:** Haipeng Wang, Junjie Wang, Peng Qi, Ximeng Yang, Kunpeng Chen, Shen Hu, Erteng Liu, Shun Zhang, Qun Gao, Rui Li, Jun Lu, Gang Deng, Daming Wang

**Affiliations:** ^1^Department of Radiology, Zhongda Hospital, Center of Interventional Radiology and Vascular Surgery, Medical School, Southeast University, Nanjing, China; ^2^Department of Neurosurgery, Beijing Hospital, National Center of Gerontology, Institute of Geriatric Medicine, Chinese Academy of Medical Sciences, Beijing, China; ^3^Graduate School of Peking Union Medical College, Beijing, China

**Keywords:** stroke, carotid artery stenting, periprocedural high-dose atorvastatin, new ischemic brain lesions, medicine management

## Abstract

**Objective:**

Carotid artery stenting (CAS) performed perioperatively with high-dose atorvastatin may reduce the incidence of new ischemic brain lesions, but more high-level evidence is needed. Furthermore, the optimal dose and course of perioperative statin therapy remain uncertain.

**Methods:**

A single-center, prospective, parallel controlled, pilot randomized clinical trial was conducted at Beijing Hospital. The study includes a total of 130 patients with CAS. The patients were randomly assigned to receive a high-dose of 80 mg/day atorvastatin (*n* = 65) or a standard-dose of 20 mg/day atorvastatin (*n* = 65) 3 days before and 3 days after planned CAS. The primary endpoint event was the cumulative incidence of silent new ischemic cerebral lesions (sNICL) on post-CAS cerebral diffusion-weighted magnetic resonance imaging (DW-MRI), transient ischemic attack (TIA), or ischemic stroke within 30 days after CAS.

**Results:**

Among the 130 patients, 123 completed the study, of which 63 were in the high-dose group and 60 were in the standard-dose group. The incidence of major endpoint events was 69.8% (44 cases) and 78.3% (46 cases) in the high-dose and standard-dose groups, respectively. There was no significant difference between the two groups (HR, 0.705; 95% CI, 0.315–1.576; *p* = 0.393). According to the stratified analysis results, the sNICL incidence was significantly different between the two groups in the symptomatic patients (HR, 0.263; 95% CI, 0.70–0.984; *p* = 0.04).

**Conclusion:**

Among patients with CAS, a periprocedural high-dose of atorvastatin did not reduce the rate of periprocedural ischemic brain damage. However, high-dose statins can reduce the incidence of sNICL after CAS in patients with symptomatic carotid stenosis.

**Clinical Trial Registration:**

Clinicaltrials.gov, identifier NCT03079115.

## Introduction

Carotid artery stenting (CAS) is an effective method to treat carotid artery stenosis. Compared with carotid endarterectomy (CEA), the trauma is milder, and the hospital stays are shorter. Nevertheless, the risk of periprocedural ischemic brain damage after the operation is higher, with 85%–90% of the cases being asymptomatic and termed silent new ischemic cerebral lesions (sNICL; Bonati et al., 2010; Cho et al., 2017). This will increase the incidence of ischemic stroke or TIA events in the future and affect the cognitive dysfunction of patients (Capoccia et al., 2010; Gensicke et al., 2015). Hence, how to reduce the risk of periprocedural ischemic brain damage in patients with CAS and further improve the safety of CAS has become the focus of recent research.

The efficacy of statins in reducing severe cardiovascular events is mainly due to the reduction in low-density lipoprotein cholesterol (LDL-C), while the lipid-lowering effect of high-dose statins is more substantial (Baigent, 2010, 2). Studies have shown that high-dose atorvastatin can effectively reduce LDL-C concentrations below 70 mg/dl, effectively reducing the incidence of major adverse cardiac and cerebrovascular events (MACCEs) in patients with ischemic stroke or TIA (Amarenco et al., 2020). Therefore, we speculate that applying high-dose statins during the perioperative period of CAS may further reduce periprocedural ischemic brain damage. The Preventing Ischemic brain damage after Carotid Artery Stenting (PICAS) pilot randomized controlled trial (RCT) was to establish the feasibility of investigating whether perioperative treatment with high-dose atorvastatin could reduce the risk of ischemic brain injury and severe adverse cardiovascular events in CAS patients.

## Materials and methods

### Trial design and oversight

This study is an extension of our previously published work (Wang et al., 2020), which outlines the study design and describes the research objectives, methodology, and overall organization. The main contribution of this manuscript over our previously published work is that it presents the obtained results and concludes this randomized controlled clinical trial.

The PICAS study was a single-center, open-label, prospective, pilot randomized controlled trial that followed the Helsinki Declaration and was approved by the Beijing Hospital Ethics Committee or Institutional Review Committee. All patients provided written informed consent. This study divided 130 patients with carotid artery stenosis who underwent stenting from August 2017 to April 2020 in the Neurosurgery Department of Beijing Hospital into a high-dose atorvastatin experimental group and a low-dose atorvastatin control group. From 3 days before CAS to 3 days after CAS, the experimental group was given 80 mg per Qd. The control group was assigned to a dose of 20 mg Qd. Both groups were treated with atorvastatin 20 mg Qd during the rest of the hospitalization. The study protocol and statistical analysis plan were published (Wang et al., 2020).

### Sample size estimates

Open-source Open-EPI v3.03 software was used for the sample size calculations in the present study. The test level α is 0.05 (two-sided), and the test efficiency power (1-β) is 0.8. Based upon findings in the abovementioned meta-analysis and the ARMYDA-9 CAROTID study, we estimated a primary endpoint incidence of 40% in routine-dose control group patients. In contrast, the incidence in the high-dose treatment group, who will undergo therapy longer than the patients in the ARMYDA-9 CAROTID study, was estimated to be lower at 15%. Given a 1:1 control to test the group ratio, it was estimated that the control group would require 56 test cases. Given a predicted 15% dropout rate, a required sample size of 65 was calculated for both groups, yielding an overall sample size of 130.

### Participants

We included patients aged 40 to 90 years who had already undergone 2 weeks of treatment with statin therapy (regardless of the type and dose of statin therapy). The detailed inclusion and exclusion criteria are shown in the published study protocol (Wang et al., 2020).

### Randomization and study treatments

All patients were enrolled in either a high-dose treatment group or a standard-dose atorvastatin group. Random number generation by a dedicated software program was then used to assign patients to the groups with 1:1 matching grouping, and no cross-grouping was permitted. The stratified blocked randomization method assigned the patients to the groups based upon age (>70 years or not) and presentation (symptomatic or asymptomatic). Symptomatic carotid stenosis was defined as ischemic stroke, TIA, or blackness in the ipsilateral cerebral hemisphere within 6 months; those lesions that did not meet the definition of appeal were considered asymptomatic carotid artery stenosis. The following interventional methods were given in this research: aspirin 100 mg/d and clopidogrel 75 mg/d were taken orally at least 3 days before treatment. The operation was performed *via* the femoral artery under local anesthesia, and blood pressure, heart rate, and blood oxygen saturation were continuously monitored. The whole body was heparinized, and the activation coagulation time was ≥250 s. An 8F vascular sheath and a guide catheter were used, and a 0.035-inch super sliding guidewire was used to deliver the guide catheter into the proper location. For a complex aortic arch and tortuous carotid artery, a coaxial catheter, guidewire exchange, and double guidewire could be used. Routine application of distal brain protection device, according to the situation, balloon preexpansion, postexpansion, or combination before and after stent placement were used to expand the stenosis. We paid attention to gently handling the guidewires and catheters during the operation to reduce the risk of plaque shedding in the arterial wall. The perioperative application of vasoactive drugs were used to maintain hemodynamic stability; the patients were fully hydrated and the amount of contrast medium was minimized. The speed of contrast injected into the body was monitored, and the balance of inflow and outflow was obtained. After the operation, the combined antiplatelet therapy of 100 mg/d aspirin and 75 mg/d clopidogrel was continued for at least 3 months, and then the treatment was changed to a single antiplatelet drug.

### Endpoint

The composite primary endpoint of major cardiovascular events is the cumulative incidence of sNICL on post-CAS cerebral diffusion-weighted magnetic resonance imaging (DW-MRI), TIA, or ischemic stroke within 30 days after CAS. The secondary endpoint events included (1) the incidence of sNICL as detected *via* cerebral DW-MRI within the period of 5 days following CAS; (2) the number of sNICL and the incidence of sNICL > 5 mm in size as detected *via* DW-MRI within this 5-day postoperative period; (3) the incidence of ischemic stroke or TIA within the 30-day postoperative period; (4) the incidence of death, any stroke, or myocardial infarction within the 30-day postoperative period; and (5) component rates of bleeding complications, as determined based upon the Thrombolysis In Myocardial Infarction criteria, and of entry-site complications (subcutaneous hematoma > 10 cm, pseudoaneurysm or arteriovenous fistula).

### Data and statistical analysis

All categorical variables are described with percentages, while continuous variables are represented in terms of means ± standard deviations. The primary analysis was performed based on the intention-to-treat (ITT) principle. Sensitivity analyses were performed for the primary endpoint event, excluding patients who did not receive a per-protocol loading dose. We performed a subgroup analysis according to age > 70 years and whether the patient was symptomatic as the protocol mentioned. The other subgroup analysis was analyzed as an exploratory analysis according to sex, dyslipidemia, side of ICA, and ulcerated plaque.

## Results

**Figure 1:**
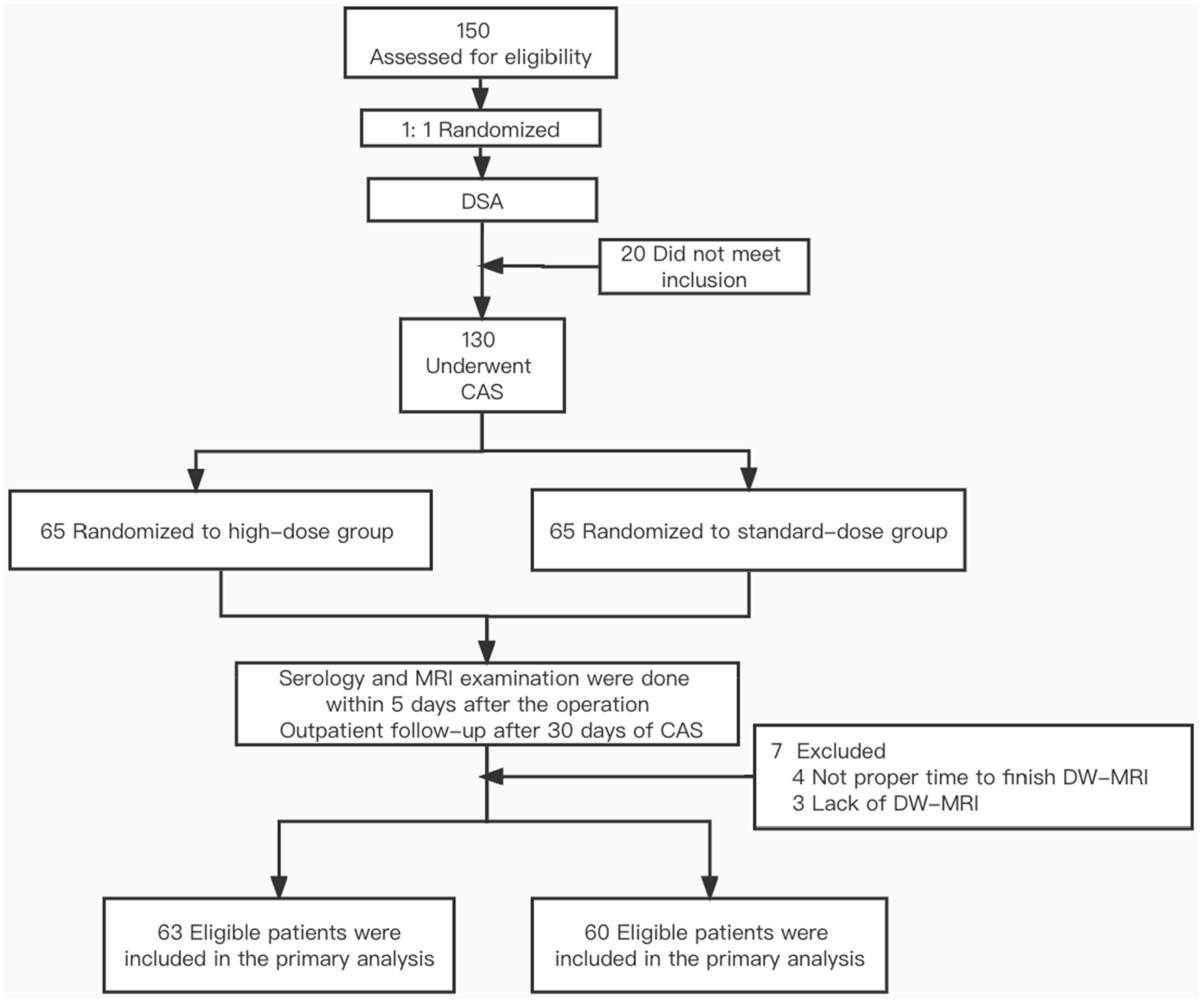
Patient enrollment and follow-up in the preventing ischemic brain damage after carotid artery stenting (PICAS) study.

From August 2017 to April 2020, 150 patients underwent randomization; 20 did not undergo CAS after the DSA examination and were excluded from the PICAS study (Figure 1). Among the 130 patients, four patients’ DW-MRI did not meet the time requirements of the protocol, and three patients lacked postoperative DW-MRI; therefore, all 7 of those patients were excluded. Additionally, 2 patients took statins for less time than the protocol requirements.

Among the 123 patients, 98 were male, and 25 were female, with an average age of 70.5 ± 8.8 years. There were 61 patients in the elderly group (age > 70) and 62 in the nonelderly group. Fifty patients had symptomatic carotid stenosis, and 73 patients were asymptomatic. Sixty-three patients were enrolled in the high-dose group, and 60 were enrolled in the standard-dose group. All patients finished follow-up. The demographic characteristics, preoperative serological data, and baseline data of the surgical conditions of the two groups are shown in Table 1. The baseline data of the two groups were similar, but there was no significant difference (*p* > 0.05). All patients used open-loop stents and remote protection devices.

**Table 1:** The baseline of patients’ characteristics, preoperative serology and surgical data.

	Standard dose group *n* = 60	High dose group *n* = 63	*P*-value
**Patients’ characteristics**			
Age	70.5 ± 1.3	70.6 ± 1.0	0.306
Male	49 (81.7%)	49 (77.8%)	0.592
Elderly patients(>70)	33 (55%)	28 (44.4%)	0.242
Symptomatic carotid stenosis	23 (38.4%)	27 (42.9%)	0.610
Smoke	16 (26.7%)	17 (27%)	0.968
Diabetes mellitus	23 (38.3%)	28 (44.4%)	0.492
Hypertension	47 (78.3)	52 (82.5%)	0.556
Dyslipidemia	30 (50%)	34 (54.0%)	0.660
Atrial fibrillation	2 (3.3%)	2 (3.2%)	0.960
Myocardial infarction	0	2 (3.2%)	0.164
Coronary intervention	12 (20%)	15 (23.8%)	0.610
Coronary artery bypass grafting	2 (3.3%)	1 (1.6%)	0.530
Oral antiplatelet drugs	31 (51.7%)	27 (42.9%)	0.640
Coronary heart disease	16 (26.7%)	18 (28.6%)	0.813
Peripheral vascular disease	12 (20.0%)	13 (20.6%)	0.930
**Preoperative serology**			
Hemoglobin	131 (123.0,144.0)	138 (127,151)	0.927
Total cholesterol	3.49 (3.005,4.195)	3.27 (2.88,4.27)	0.894
LDL-C	1.99 (1.655,2.570)	1.82 (1.51,2.75)	0.947
Alanine transaminase	17 (11.0,24.0)	18.0 (14.0,25.5)	0.921
Aspartate transaminase	19 (16.0,23.0)	18.0 (15.0,22.0)	0.993
Creatine kinase	77 (61.5,105.5)	78 (53.5,108.0)	0.765
Lactate dehydrogenase	157 (135.5,179.0)	171 (158,187.5)	0.696
Total bilirubin	10 (7.3,14.5)	11.15 (9.25,13.925)	0.416
Serum creatinine	78 (66,97)	76 (64.5,94)	0.823
Serum urea	5.84 (4.98,7.48)	5.79 (5.28.7.01)	0.907
Uric acid	368 (284,422)	329.0 (281.0,405.0)	0.974
**Surgical data**			
Side: left	27 (45%)	36 (57.1%)	0.178
Lesion length (mm)	15.88 (11.35,23.06)	16.25 (10.43,21.53)	0.636
Normal diameter of distal stenosis (mm)	5.505 (4.72,6.53)	5.57 (4.77,6.65)	0.927
Ulcer plaque (*n*, %)	32 (50.8%)	36 (60%)	0.256
Pre-dilatation (*n*, %)	56 (93.3%)	56 (88.9%)	0.388
Post-dilatation (*n*, %)	34 (56.7%)	28 (44.4%)	0.175
Operation duration (*n*, %)	91 (75,120)	85 (65,120)	0.246
Degree of stenosis (DSA)	76 (68.25,85.75)	70 (60,85)	0.107
Degree of residual stenosis (DSA)	5.5 (0,19)	5 (0,14)	0.927
Type III aortic arch	8 (13.3%)	8 (12.7%)	0.917

The primary endpoints mainly adopted the ITT principle. Three patients lacked major endpoint events and four patients failed to complete DW-MRI within the required time; therefore, those seven patients were excluded from the statistical total analysis set. Among 123 patients, the total incidence of major endpoint events was 73.2% (90/123). The incidence of major endpoint events in the high-dose group was 69.8% (44/63), among which 42 patients showed sNICL on DW-MRI after the operation; the incidence was 66.7% (42/63). In the high-dose group, 2 patients suffered from ischemic stroke during the perioperative period, 1 patient suffered from retinal embolism during the operation, and the other patient suffered from mild hemiplegia on the second day after the operation. In the standard-dose group, the incidence of major endpoint events was 78.3% (46/60), of which 45 patients showed sNICL on DW-MRI, and the incidence was 75% (45/60). One patient in the standard-dose group developed mild hemiplegia immediately after the operation. The absolute difference in the incidence of major endpoint events between the two groups was 8.5% (HR, 0.705; 95% CI, 0.315–1.576; *p* = 0.393). The *p*-values were not significant for the secondary endpoints (Table 2).

**Table 2:** Hazard ratios for clinical end points.

End points	Standard dose group (n = 60)	High dose group (*n* = 63)	HR (95%CI)	*P*-value
**Primary end points (%)**				
The cumulative incidence of sNICL on post-CAS cerebral DW-MRI, TIA or ischemic stroke within 30 days after CAS	46 (78.3%)	44 (69.8%)	0.705 (0.315–1.576)	0.393
**Secondary end points (%)**				
The incidence of sNICL in DW-MRI after CAS	45 (75%)	42 (66.7%)	0.667 (0.304–1.461)	0.310
The incidence of ipsilateral sNICL	30 (50%)	36 (57.1%)	1.333 (0.655–2.714)	0.427
The number of sNICL	2.5 (0.25,7.75)	3 (0,7)		0.902
The incidence of sNICL > 5 mm	24 (40%)	28 (44.4%)	1.2 (0.586–2.457)	0.618
The incidence of severe adverse cardiovascular events within 30 days after CAS	1 (1.7%)	2 (3.2%)	1.934 (0.171–21.907)	0.588
Component rates of bleeding complications	2 (3.3%)	1 (1.6%)	0.468 (0.041–5.297)	0.530

**Figure 2:**
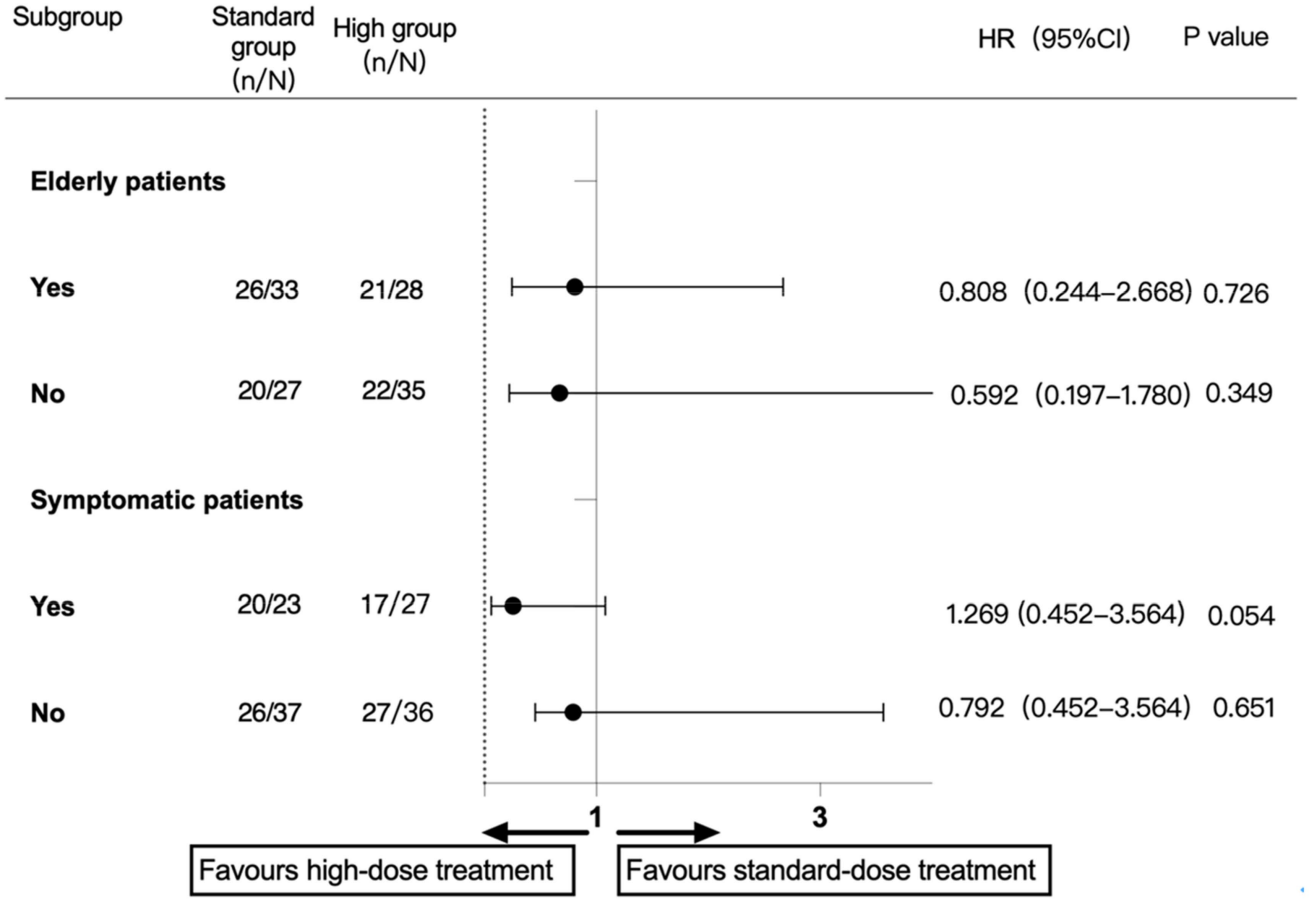
Subgroup analysis of the component primary outcome.

**Figure 3:**
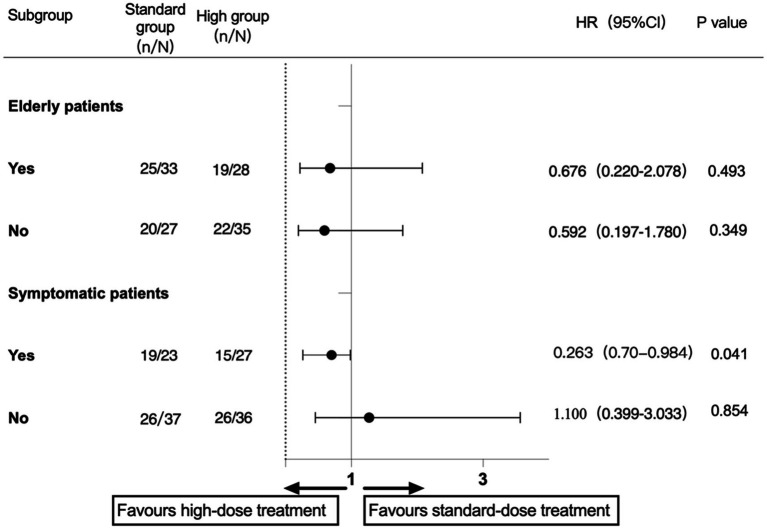
Subgroup analysis of the sNICL.

A subgroup analysis was performed according to the prestratification of the protocol (Figures 2, 3). In the patients with symptomatic carotid stenosis, the incidence of primary endpoint events was 74% (37/50), of which the incidence in the high-dose group was 63% (17/27) and that in the standard-dose group was 87% (20/23; HR, 0.255; 95% CI, 0.060–1.080; *p* = 0.054). In the patients with asymptomatic carotid stenosis, the incidence of primary endpoint events was 72.6% (53/73), 75% (27/36) in the high-dose group, and 70.3% (26/37) in the standard-dose group. There was no significant difference in the incidence of major endpoint events between the two groups (HR, 0.792; 95% CI, 0.452–3.564; *p* = 0.651). Among the elderly patients, the incidence of primary endpoint events was 77% (47/61), of which the incidence in the high-dose group was 44.7% (21/28) and that in the low-dose group was 55.3% (26/33). There was no significant difference between the two groups (HR, 0.808; 95% CI, 0.244, 2.668; *p* = 0.726). The incidence of primary endpoint events in the nonelderly patients was 69.4% (43/62), among which the incidence in the high-dose group was 62.9% (22/35) and that in the standard-dose group was 74.1% (20/27). There was no significant difference between the two groups (HR, 0.671; 95% CI, 0.221, 2.032; *p* = 0.479). Regarding the incidence of sNICL, the incidence of the high-dose group was 71.4% (20/28) and that of the standard-dose group was 75.8% (25/33). There was no significant difference between the two groups (HR, 0.800; 95% CI, 0.255, 2.509; *p* = 0.702). In the nonelderly group, there was an incidence of sNICL of 62.9% (22/35) in the high-dose group, it was 74.1% (20/27) in the low-dose group, and these were not significantly different (HR, 0.592; 95% CI, 0.197, 1.780; *p* = 0.349).

Sensitivity analyses were performed based on per-protocol (PP) analysis for the primary outcome, and patients who did not receive a loading dose as protocol request were excluded. In our study, one of the patients in each group did not meet the requirements of the PICAS protocol. The PP analysis showed that the incidence of primary endpoint events in the high-dose group was 69.4% (43/62), while that in the standard-dose group was 76.3% (45/59; HR, 0.704; 95% CI, 0.314–1.578; *p* = 0.393). The incidence of sNICL in the high-dose and standard-dose groups was 66.1% (41/62) and 74.6% (44/59), respectively. Overall, the results of the sensitivity analyses were consistent with the study’s main findings.

**Figure 4:**
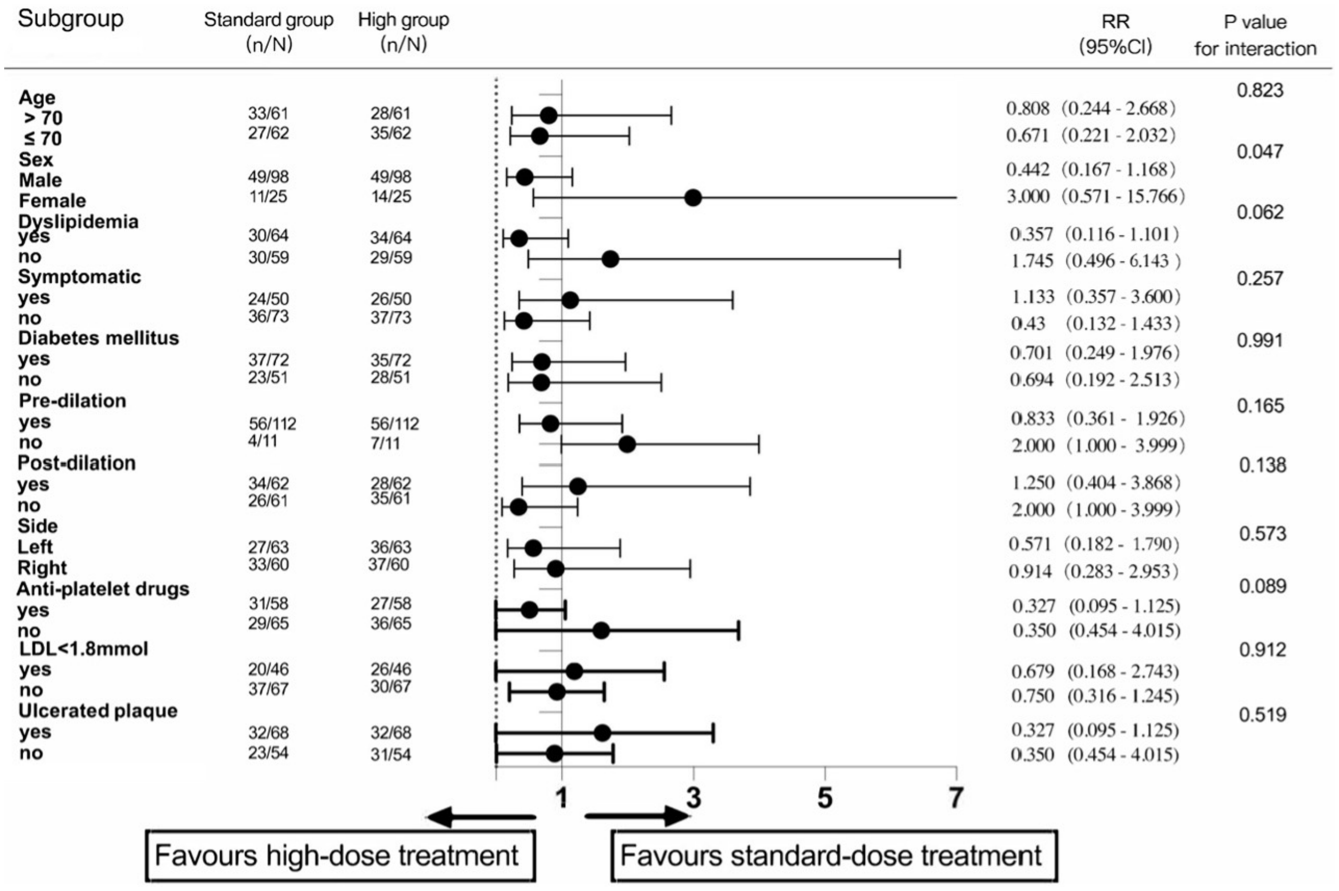
Exploratory analysis in different subgroups.

We also performed an exploratory analysis of the influence of the different subgroups on the primary endpoint events (Figure 4). The results showed that different sexes might have opposite trends in high-dose statin treatment (*p*-value for the interaction = 0.047).

None of the patients complained of adverse reactions related to statins during follow-up. Before and after the operation, the serological examination showed that total cholesterol, low-density lipoprotein, alanine transaminase, blood urea, blood urea, and blood uric acid decreased, creatine kinase and lactate dehydrogenase had no apparent changes, and serum creatinine and total bilirubin increased slightly. Nevertheless, they were all within the normal reference value range.

## Discussion

Statins are the cornerstone of drug treatment for patients with atherosclerotic diseases (ASCVDs) and can prevent cardiovascular events. It has been reported that the main mechanism of action for statins is to reduce low-density lipoprotein cholesterol, and the early pleiotropic effect of statins after initiation also has a protective effect on cardiovascular events (Patti et al., 2013). At present, the effect of statins on ischemic brain injury during the CAS perioperative period needs to be proven by higher-level research.

The PICAS pilot RCT explored the effect of perioperative high-dose atorvastatin on new ischemic lesions in the CAS perioperative period, and, due to the study’s experimental nature, the research conclusion is more credible than that of previous observational studies. To the best of our knowledge, this study is mainly aimed at the Asian population and is the first RCT study on the Chinese people. The ARMYDA-9 CAROTID study shows that (Patti et al., 2013) preoperative high-dose atorvastatin (120 mg) can reduce the occurrence of early cerebral ischemia events in European and American countries. Due to ethnic differences in the polymorphisms of the genes of statin-dependent metabolic enzymes and transporters, there are differences in the drug response and safety between Chinese, European, and American populations. Several guidelines indicate that Asian patients may need careful monitoring when using high-intensity statins, which affects their dosing intensity when starting statins (Stone et al., 2014). However, the 2016 Guidelines for the Prevention and Treatment of Dyslipidemia in Adults in China pointed out that the potential increase in benefit and the safety of the maximum allowable dose of statins in the Chinese population have not yet been determined (Lloyd-Jones et al., 2016).

A retrospective cohort study in Japan showed that the oral administration of 20 mg rosuvastatin or 40 mg atorvastatin before surgery can reduce the positive rate of ischemic lesions on DW-MRI after the procedure (Shinozaki et al., 2020). Another prospective cohort showed that taking pitavastatin for 4 weeks before surgery can reduce perioperative ischemic complications (Takayama et al., 2014). However, there were significant differences in total cholesterol and LDL-C baseline values between the treatment and control groups in both studies, with the high-dose group having values that were significantly lower than the control group. In the retrospective cohort study, the usage and dosage of statins in the control group were not mentioned. Therefore, the evidence level of these studies is not high, and the difference in the baseline data reduces the applicability of their research results, which has certain limitations.

The results of this study showed that perioperative high-dose atorvastatin did not reduce the incidence of the primary component outcome within 30 days. However, in patients with symptomatic carotid stenosis receiving CAS, the perioperative administration of high-dose atorvastatin can reduce sNICL on DW-MRI and may reduce the cumulative incidence of sNICL on post-CAS cerebral DW-MRI, TIA or ischemic stroke within 30 days after CAS, since the results show a strong tendency toward statistical significance (*p* = 0.054).

There was no significant difference in the baseline data among the patients in this study, and no statin-related adverse reactions occurred during the 30-day follow-up. Before and after the operation, the serological examinations showed no abnormal changes in the liver and kidney function indexes, which proved the safety of perioperative atorvastatin and verified our research design. In this study, the total incidence of ischemic events within 30 days was 73.2%, and the incidence of sNICL was 66.7%, which is consistent with previous studies (Müller et al., 2019; Volkers et al., 2019). Nevertheless, compared with recent literature reports, the incidence of sNICL in this study was higher. We consider the following reasons. First, it may be related to our detailed cerebral angiography. Diagnostic angiography can make the incidence of new ischemic lesions in DWI-MRI as high as 24.65% (Al-Hussain et al., 2021). In our study, each subject underwent bilateral internal carotid, vertebral and subclavian arteriography and selective internal carotid angiography. Repeated attempts to push the catheter and guidewire may increase the shedding of thrombus fragments and cause an embolism at the distal end. However, foreign studies usually do not perform cerebral angiography and selective internal carotid angiography. Second, this study’s proportion of male patients was as high as 80%. Some studies have reported that male patients may have an increased incidence of new ischemic lesions after CAS (Pini et al., 2013). In addition, the average age of the subjects in this study (70.54 ± 8.757) was older than that of other studies. Many studies have shown that new ischemic lesions after CAS increase with age. Third, all patients in this study received MRI with a 3.0 T field strength. There are differences in the detection rate of DWI ischemic lesions by MRI with different field strengths. In other studies, the accuracy of detecting new ischemic foci by high-field magnetic resonance is higher than that of 1.5 T field magnetic resonance. It is easier to find new ischemic foci (Bijuklic et al., 2013a,b). Fourth, the subjects in this study underwent MRI within 1–5 days after CAS, while in most other studies, MRIs were completed within 12 to 48 h after CAS. There is evidence that microemboli fragments may still be produced within a few weeks after stent placement, leading to new ischemic foci (Jiang et al., 2011). Therefore, the reexamination of MRI 1–5 days after the operation, as designed in this study, can identify the incidence of NICL after the operation more accurately. Fifth, in all the patients in this study, open-loop stents and distal protective devices were used, and it has been reported that open-loop stents (Wodarg et al., 2018; de Vries et al., 2019) and distal embolization protective devices (Schmidt et al., 2004; Bijuklic et al., 2012) may be risk factors leading to asymptomatic new ischemic brain injury during the perioperative period. Therefore, these factors may lead to a higher incidence of new ischemic focus events in our study than in other studies.

According to the subgroup analysis specified in the PICAS protocol, perioperative high-dose atorvastatin can reduce early sNICL after CAS in patients with symptomatic carotid stenosis, which provides a reliable basis for the application of high-dose atorvastatin in the perioperative period. The exploratory analysis of this study found that high-dose atorvastatin has different influence trends on major endpoint events in different sexes (*p* = 0.047). We consider that this subgroup has certain limitations, including that it does not meet the principle of randomness and that the sample size is small. Therefore, such results need to be carefully interpreted, and a further clinical trial basis is required to support clinical evidence.

Compared with the ARMYDA-9 CAROTID randomized controlled study [8], the PICAS study did not conclude that intensive lipid-lowering therapy can reduce the risk of NICL after surgery in all patients, which is inconsistent with its results. In addition to the influence of MRI examination time and the operation factors mentioned above, the higher proportion of symptomatic carotid stenosis patients, ulcer plaque, and intraoperative predilation included in the PICAS study is also an important reason. Comparing the proportion of those three variables in the ARMYDA-9 CAROTID study to the PICAS study, the data were 14.1% vs. 40.6%, 29.5% vs. 55.3%, and 19.9% vs. 91.1%, respectively. Previous studies have shown that symptomatic carotid stenosis, ulcer plaque, and predilation are independent risk factors for new ischemic foci during the perioperative period (Rots et al., 2019).

### Conclusion

The PICAS pilot study does not support the routine use of high-dose atorvastatin in unselected CAS patients. However, in symptomatic carotid stenosis patients, perioperative high-dose atorvastatin treatment can probably reduce the incidence of sNICL during the perioperative period and possibly reduce the incidence of ischemic events within 30 days after CAS. In summary, this study explores the best statin management for CAS patients during the perioperative period. Perioperative statin therapy in CAS is safe, and the risk of adverse events is not increased. Larger multiple-centers randomized clinical trials are needed to investigate whether perioperative high-dose atorvastatin has efficacy in preventing the onset of ischemic events after CAS.

### Study limitations

There are some limitations in this study. The specification of the type of statins being used by patients was not strictly required before admission, which may have had a specific impact on the perioperative results. Other baseline data, such as carotid artery tortuosity, other intracranial and extracranial artery stenosis, balloon dilatation time and pressure, and DW-MRI reexamination time, were not considered in the two groups. This study was a single-center study, and the number of CAS operators was limited to 3. However, each operator has more than 10 years of interventional experience (at least 50 cases/year). According to the condition, the surgeon selects specific operations such as intraoperative brain protection devices, stent and balloon type, preexpansion, and postexpansion. Therefore, we cannot rule out that these related factors may cause a higher incidence of new ischemic cerebral ischemia injury and stroke events during the perioperative period, thus masking the protective effect of high-dose atorvastatin. Besides, as this investigation was designed as a pilot, it enabled us to calculate that a reasonable sample size would allow an efficient power of relevant study endpoints. A subsequent larger multicenter study will design based on the enrolment rate, protocol compliance, and relevant events rate that this pilot trial established.

## Data availability statement

The datasets presented in this study can be found in online repositories. The names of the repository/repositories and accession number(s) can be found in the article/supplementary material.

## Ethics statement

The studies involving human participants were reviewed and approved by Beijing Hospital Ethics Committee. The patients/participants provided their written informed consent to participate in this study.

## Author contributions

HW, JL, DW, and JW contributed to the design and conception of the study. HW, JW, PQ, XY, KC, SH, SZ, EL, RL, QG, JL, GD, and DW contributed to the acquisition of data and analysis interpretation of the results. HW, JL, and DW drafted the first version of the manuscript. All authors contributed to the article and approved the submitted version.

## Funding

The trial was funded by National High Level Hospital Clinical Research Funding with grant numbers (121-2016006 and 121-2018086), the Non-profit Central Research Institute Fund of the Chinese Academy of Medical Sciences (no. 2019TX320002), and CAMS Innovation Fund for Medical Sciences (CIFMS; grant number 2021-2M-C&T-B-092).

## Conflict of interest

The authors declare that the research was conducted in the absence of any commercial or financial relationships that could be construed as a potential conflict of interest.

## Publisher’s note

All claims expressed in this article are solely those of the authors and do not necessarily represent those of their affiliated organizations, or those of the publisher, the editors and the reviewers. Any product that may be evaluated in this article, or claim that may be made by its manufacturer, is not guaranteed or endorsed by the publisher.
